# Intestinal Dysbiosis and Risk of Posttransplant Clostridioides difficile Infection in a Longitudinal Cohort of Liver Transplant Recipients

**DOI:** 10.1128/msphere.00361-22

**Published:** 2022-09-22

**Authors:** Angela Gomez-Simmonds, Medini K. Annavajhala, Maria Patricia Nunez, Nenad Macesic, Heekuk Park, Anne-Catrin Uhlemann

**Affiliations:** a Division of Infectious Diseases, Columbia University Irving Medical Center, New York, New York, USA; b Department of Microbiology & Immunology, Columbia University, New York, New York, USA; c Department of Infectious Diseases, The Alfred Hospital and Central Clinical School, Monash University, Melbourne, Victoria, Australia; d Centre to Impact AMR, Monash University, Melbourne, Victoria, Australia; University of Michigan-Ann Arbor

**Keywords:** 16S rRNA sequencing, *Clostridium difficile*, intestinal microbiome, liver transplantation

## Abstract

Clostridioides difficile infection (CDI) has a higher incidence in solid organ transplant recipients than other hospitalized patients and can lead to poor outcomes. Perturbations to the intestinal microbiome are common in patients undergoing liver transplant (LT); however, the impacts of microbial diversity and composition on risk of CDI in this patient population is incompletely understood. Here, we assessed patients in an established, longitudinal LT cohort for development of CDI within 1 year of transplant. Clinical data were compared for patients with and without CDI using univariable models. 16S rRNA sequencing of fecal samples was performed at multiple pre- and posttransplant time points to compare microbiome α- and β-diversity and enrichment of specific taxa in patients with and without CDI. Of 197 patients who underwent LT, 18 (9.1%) developed CDI within 1 year. Pre-LT Child-Pugh class C liver disease, postoperative biliary leak, and use of broad-spectrum antibiotics were significantly associated with CDI. Patients who developed CDI had significantly lower α-diversity than patients without CDI overall and in samples collected at months 1, 3, and 6. Microbial composition (β-diversity) differed between patients with and without CDI and across sampling time points, particularly later in their posttransplant course. We also identified 15 (8%) patients with toxigenic C. difficile colonization who did not develop CDI and may have had additional protective factors. In summary, clinical and microbiome factors are likely to converge to impart CDI risk. Along with enhanced preventive measures, there may be a role for microbiome modulation to restore microbial diversity in high-risk LT patients.

**IMPORTANCE** Liver transplant (LT) recipients have high rates of Clostridioides difficile infection (CDI), which has been associated with poor outcomes, including graft-related complications and mortality, in prior studies. Susceptibility to CDI is known to increase following perturbations in intestinal commensal bacteria that enable germination of C. difficile spores and bacterial overgrowth. In LT patients, changes in the intestinal microbiome resulting from advanced liver disease, surgery, and other clinical factors is common and most pronounced during the early posttransplant period. However, the relationship between microbiome changes and CDI risk after LT remains unclear. In this study, we investigated clinical and microbiome factors associated with development of CDI within the first year after LT. The importance of this work is to identify patients with high-risk features that should receive enhanced preventive measures and may benefit from the study of novel strategies to reconstitute the intestinal microbiome after LT.

## INTRODUCTION

Clostridioides difficile infection (CDI) is the most common cause of hospital-associated infectious diarrhea, with a particularly high burden of disease occurring among patients undergoing solid organ transplantation (SOT) (1). In this patient population, CDI rates of 2 to 15% have previously been reported (2–5), reflecting a 5-fold higher incidence compared to other adult hospitalized patients (1). Infection may lead to poor outcomes, including graft-related complications, prolonged hospital stay, need for colectomy, and mortality (1–3, 6). However, the mechanisms underlying increased CDI risk in SOT recipients are not fully clear and may differ from those in nontransplant patients.

Immune dysregulation and perturbations to intestinal microbial diversity and community structure in the setting of chronic illness may be exacerbated by peritransplant induction of immunosuppression, antibiotic prophylaxis, and surgery, leading to high rates of CDI in the early posttransplant period. Frequent hospital exposure, antibiotic use, and increased immunosuppression due to rejection episodes may confer ongoing risk. In patients undergoing liver transplantation (LT), direct surgical manipulation of the gastrointestinal tract and biliary system complications may be additional contributors to both early and later infection (7).

We previously reported changes in microbial diversity and composition from pretransplant to 1-year posttransplant in a longitudinal cohort of LT patients (8). 16S rRNA sequencing of serial fecal samples revealed various temporal dynamics in microbiome α- and β-diversity before transplant and during recovery, based on underlying liver disease etiology, posttransplant complications, antibiotic use, and other clinical factors. Colonization with multidrug-resistant organisms (MDROs) such as carbapenem-resistant *Enterobacteriaceae* and vancomycin-resistant *Enterococci* was significantly associated with reductions in Shannon α-diversity and alterations in microbial composition including loss of protective taxa and enrichment of potentially pathogenic taxa, with differences in onset and persistence by organism and resistance profile (9). While the presence of these quantitative and/or qualitative changes in intestinal microbiome community structure compared to appropriate control populations, referred to here as dysbiosis, has the potential to influence other posttransplant infections, the relationship between pre- and posttransplant microbial composition and development of CDI has not been determined.

CDI is well known to be a microbiome-related disease, with numerous studies linking antibiotic-associated perturbations in intestinal microbiota to the development and recurrence of CDI (10–16). Moreover, fecal microbial transplant (FMT), which leads to increased α-diversity in the recipient with microbial communities resembling those of the donor (17, 18), has been established as a successful therapeutic modality (19). CDI risk in SOT recipients has been shown to vary over time, with the highest risk occurring in the early posttransplant period (within 1 week to 1 month posttransplant) (5, 20). Previous studies have also suggested increased risk of later infection in liver compared to other organ transplant recipients (5). For this study, we hypothesized that risk of posttransplant CDI is associated with changes in intestinal microbial diversity and composition occurring before and after LT and further modulated by other clinical risk factors. Thus, we comprehensively studied clinical and microbiome parameters in the 1 year following LT to better understand how they may affect posttransplant development of CDI in LT recipients.

## RESULTS

### Clinical characteristics of LT patients with CDI.

**FIG 1:**
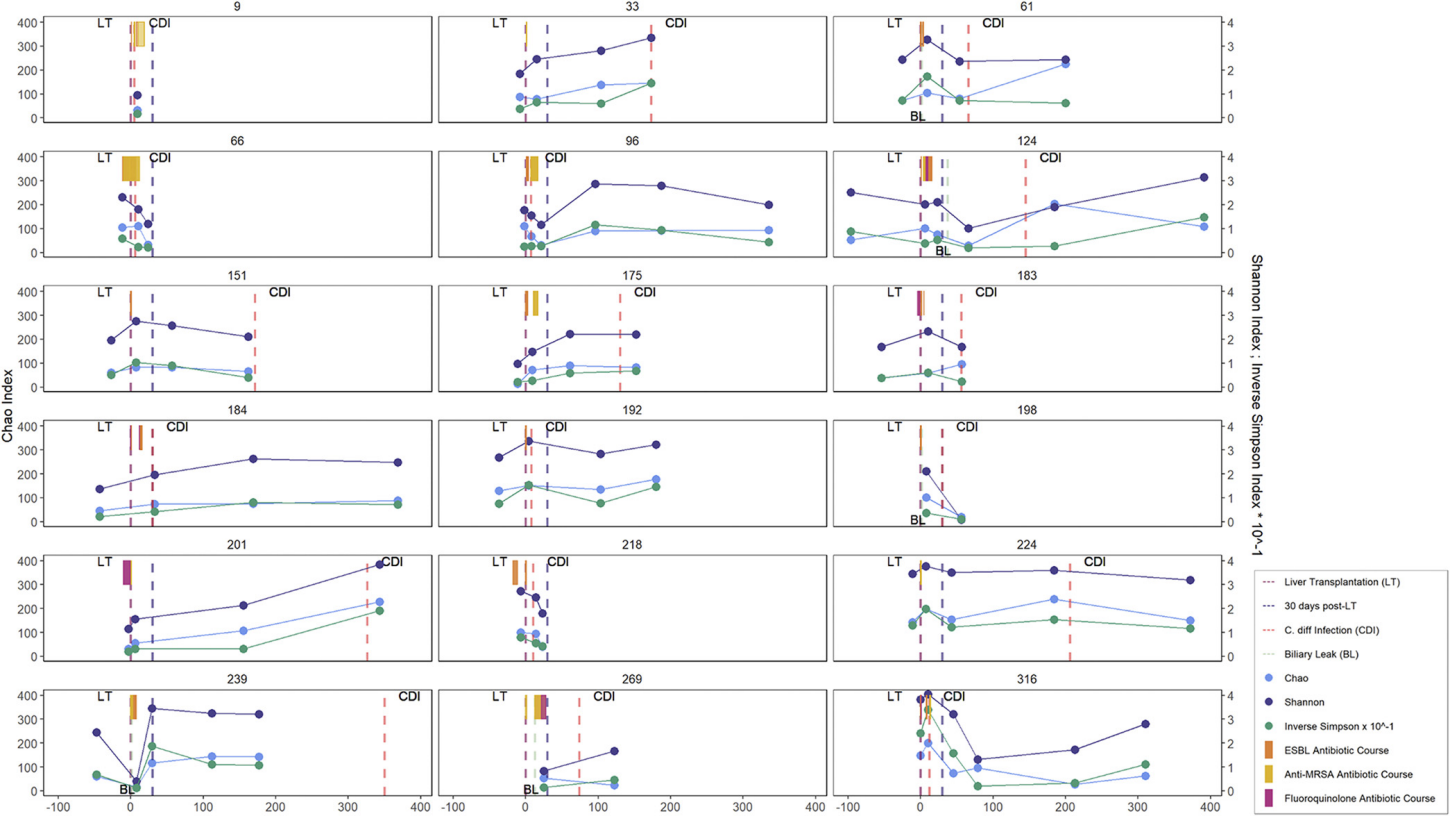
α-Diversity (Chao, Shannon, Inverse Simpson × 10^−1^) of longitudinal fecal samples collected from patients with development of C. difficile infection (CDI) within 1 year after liver transplantation (LT). For each patient with CDI, the longitudinal trajectory of α-diversity values for available stool samples for each collection time point is indicated, as are the time to development of CDI (red dashed line), biliary leak (BL, light green dashed line), and inpatient antibiotic exposure (shown by antibiotic class; ESBL, extended-spectrum beta-lactamase; MRSA, methicillin-resistant *Staphylococcus aureus*). Overall, CDI did not appear to coincide with proximate declines in α-diversity values for most patients.

There were 197 enrolled patients who underwent LT during the study period and met criteria for inclusion in the analysis (8). Of these, 18 (9.1%) developed CDI within 1 year posttransplant. Five patients, including two of those with CDI posttransplant, also had a documented episode of CDI prior to LT. The number of days from LT to posttransplant development of CDI varied widely from 5 to 350 days (median 61; Fig. 1). In 8 of 18 (44%) patients, CDI developed within the early posttransplant period (defined here as within 30 days of LT); three cases occurred within 1 week of transplant.

Among patients with CDI, 7 of 18 (39%) met criteria for severe CDI based on recent clinical guidelines (21). Few patients developed fever (6 of 18 [33%] patients had maximum temperatures >38°C) or leukocytosis (3 of 18 [17%] had white blood cell [WBC] counts ≥15,000 cells/mL) within 48 h of positive testing for C. difficile. Notably, 5 of 18 (28%) patients had WBC counts ≤3,500 cells/mL, and 3 of those 5 patients had an absolute neutrophil count <1,000 cells/mL. Two patients were hypotensive within 48 h and were considered to have fulminant disease. None developed severe complications such as ileus, toxic megacolon, need for colectomy, or death at the time of the first posttransplant episode of CDI. However, one patient who did not meet criteria for severe CDI initially was readmitted approximately 2 months later with persistent diarrhea and acute renal failure and found to have pseudomembranous colitis (confirmed by colonoscopy and compatible histopathologic findings). The patient underwent subtotal colectomy but ultimately expired. Two additional patients had recurrent CDI within 1 year after LT.

The demographics were similar between patients who developed CDI within 1 year versus patients without posttransplant CDI (Table 1). NAFLD was the most common indication for transplantation in patients with CDI (5 of 18, 28%) compared to HCV in CDI-negative patients (73 of 179, 41%). However, liver disease etiology did not significantly differ by CDI status. Patients who went on to develop CDI had significantly higher Child-Pugh scores (median 11 [interquartile range (IQR) 3] versus 9 [IQR 4]; *P = *0.02); 72% of patients with CDI had Child-Pugh class C liver disease compared to only 43% of CDI-negative patients (*P = *0.03). Postoperative biliary leak was also significantly associated with CDI (28% versus 8%; *P = *0.02; see Fig. 1 for event times in patients with CDI). Last, patients in both groups had extensive antibiotic exposure during their initial hospitalization for LT. Although the likelihood of rehospitalization after the LT hospitalization was similar, aggregate antibiotic use was significantly higher in patients with CDI during subsequent hospitalizations (median 15 [IQR 15] versus 12 [IQR 19] aggregate days of antibiotic use; *P = *0.0002), including increased exposure to broad-spectrum antibiotics. These risk factors are likely to be interconnected, as shown in a directed acyclic graph demonstrating potential pathways whereby these variables may influence the causal relationship between LT and CDI (Fig. S1).

**TABLE 1:** Comparison of clinical parameters in LT patients with and without CDI during the first year posttransplant^*a*^

Variable	CDI (*n* = 18)	No CDI (*n* = 179)	*P* value
Patient demographics and comorbidities			
Age at LT, median (IQR)	56 (17)	60 (11)	0.07
Male sex, *n* (%)	13 (68)	113 (63)	0.8
LT indication, *n* (%)			
HCV	4 (22)	73 (41)	0.2
HBV	2 (11)	8 (4)	
NAFLD	5 (28)	28 (16)	
ARLD	1 (6)	20 (11)	
Biliary disease^*b*^	3 (17)	20 (11)	
AIH	3 (17)	11 (6)	
Other	0	19 (11)	
HCC, *n* (%)	3 (17)	72 (40)	0.09
Living donor, *n* (%)	4 (22)	28 (16)	0.5
CCI score, median (IQR)	5 (3)	6 (2)	0.1
MELD-Na, median (IQR)	18 (15)	16 (9)	0.2
Child-Pugh score, median (IQR)	11 (3)	9 (4)	0.02^*c*^
Child-Pugh class C, *n* (%)	13 (72)	77 (43)	0.03^*c*^
Transplant hospitalization clinical course and complications			
Reoperation, *n* (%)	10 (56)	75 (42)	0.4
Postoperative bleeding, *n* (%)	2 (11)	47 (26)	0.3
Biliary leak, *n* (%)	5 (28)	14 (8)	0.02^*c*^
Biliary stricture, *n* (%)	2 (11)	31 (17)	0.7
ICU readmission, *n* (%)	7 (39)	44 (25)	0.3
Duration of hospitalization in days, median (IQR)	17 (7)	13 (13)	0.2
Complications and outcomes during the first yr after LT			
Rehospitalization, *n* (%)	11 (61)	96 (54)	0.7
Rejection episode, *n* (%)	6 (33)	47 (26)	0.6
Death, *n* (%)	2 (11)	8 (5)	0.2
Antibiotic exposure			
Aggregate days of inpatient antibacterial use during the initial transplant hospitalization, median (IQR)	15 (15)	12 (19)	0.7
Antibacterial exposure by drug class during the initial transplant hospitalization, *n* (%)			
Narrow-spectrum β-lactam^*d*^	4 (22)	35 (20)	0.8
Broad-spectrum β-lactam^*e*^	17 (94)	160 (89)	0.7
Fluoroquinolone	6 (33)	26 (15)	0.08
Anti-MRSA antibiotic^*f*^	11 (61)	77 (43)	0.2
Aggregate days of inpatient antibacterial use during subsequent hospitalization(s), median (IQR)	40 (48)	3 (17)	0.0002^*c*^
Antibacterial exposure by drug class during subsequent hospitalization(s), *n* (%)			
Narrow-spectrum β-lactam^*d*^	4 (22)	19 (11)	0.2
Broad-spectrum β-lactam^*e*^	14 (78)	73 (41)	0.006^*c*^
Fluoroquinolone	11 (61)	63 (35)	0.056
Anti-MRSA antibiotic^*f*^	15 (83)	52 (29)	<0.0001^*c*^

a CDI, C. difficile infection; LT, liver transplantation; IQR, interquartile range; HCV, hepatitis C virus; HBV, hepatitis B virus; ARLD, alcohol-related liver disease; NAFLD, nonalcoholic fatty liver disease; AIH, autoimmune hepatitis; HCC, hepatocellular carcinoma; MELD-Na, model of end-stage liver disease with sodium; ICU, intensive care unit; CCI, Charlson Comorbidity Index; MRSA, methicillin-resistant Staphylococcus aureus.

b Biliary disease classification includes primary sclerosing cholangitis, primary biliary cirrhosis, chronic cholangitis, biliary atresia, and other diseases of the biliary tract requiring transplant.

*^c^*Meets criteria for statistical significance with *P* < 0.05.

d Penicillin, ampicillin, first- or second-generation cephalosporin, or aztreonam.

e β-Lactam/β-lactamase inhibitor combination antibiotic, third- or fourth-generation cephalosporin, or carbapenem.

f Vancomycin, linezolid, or daptomycin.

10.1128/msphere.00361-22.1 FIG S1 Directed acyclic graph (DAG) demonstrating possible causal relationships between liver transplantation (LT), C. difficile infection (CDI), and including clinical variables that were significantly associated with development of CDI after LT. We hypothesize that antibiotic exposure and biliary leak mediated the causal relationship between LT and CDI, while Child-Pugh class C liver disease was a confounder. Download FIG S1, TIF file, 1.6 MB. Copyright © 2022 Gomez-Simmonds et al. 2022 Gomez-Simmonds et al. https://creativecommons.org/licenses/by/4.0/ This content is distributed under the terms of the Creative Commons Attribution 4.0 International license.

### Intestinal microbial α-diversity and CDI.

We analyzed 646 longitudinal fecal samples provided by patients in our LT cohort at specified time points (pretransplant, posttransplant week 1, month 1, month 3, month 6, and month 12). This included 70 samples collected from 18 patients with CDI (Fig. 1), most of whom (15 of 18, 83%) had samples collected within 30 days of positive testing for C. difficile. Across all samples, 6,634 amplicon sequence variants (ASVs) were identified; 3,117 (47%) of these were identified in at least 5% of samples within the collection, while the remainder were singletons.

**FIG 2:**
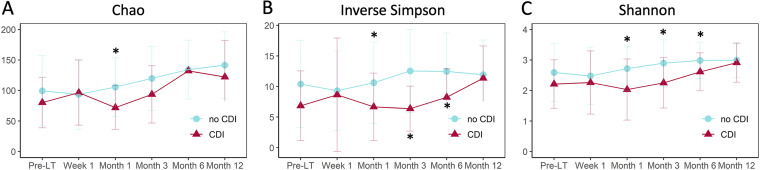
Mean α-diversity (Chao, Inverse Simpson, Shannon) at specific sampling time points from pretransplant to 1 year posttransplant in fecal samples from liver transplantation (LT) in patients with and without C. difficile infection (CDI). (A to C) Chao (A), Inverse Simpson (B), and Shannon (C) α-diversity was significantly lower in patients with posttransplant CDI at month 1. For Inverse Simpson and Shannon index values only, the differences remained statistically significant at months 3 and 6; the values then converged during the 1 year posttransplant by all indices. *, *P* < 0.05.

Overall, LT patients with and without CDI demonstrated a decrease in intestinal microbiome α-diversity (both species richness and relative distribution) posttransplant followed by a gradual increase to above pretransplant levels over the 1 year posttransplant period, based on all three α-diversity indices analyzed in this study (Chao, Inverse Simpson, and Shannon; Fig. 2). However, the time of CDI diagnosis did not necessarily correspond to decreasing or nadir α-diversity, as patients developed CDI at a range of α-diversity index values (Fig. 1).

Across all sampling time points, intestinal microbial α-diversity was significantly lower in patients with compared to without posttransplant CDI using the Chao, Inverse Simpson, and Shannon indices (mean [standard deviation (SD)] 96.1 [53.4] versus 112.9 [55.5], *P = *0.02; 7.8 [6.2] versus 11.0 [6.6], *P = *0.0001; and 2.3 [0.9] versus 2.8 [0.8], *P = *0.0001; respectively), indicating differences in both richness and evenness between groups. We also detected significant differences in α-diversity at individual time points in patients who developed CDI within 1 year after LT compared to controls (Fig. 2). Pretransplant and week 1 α-diversity did not significantly differ between groups. However, α-diversity was significantly lower in patients with posttransplant CDI at month 1 using all three indices (71.9 [36.1] versus 105.6 [47.9], *P = *0.01; 6.7 [5.5] versus 10.6 [6.6], *P = *0.03; and 2.0 [1.0] versus 2.7 [0.7], *P = *0.001; respectively). For Inverse Simpson and Shannon index values only, the differences remained statistically significant at months 3 and 6, suggesting increases in the number of species present (as reflected by the Chao index) preceded sample evenness (Inverse Simpson). However, by month 12 posttransplant, Chao, Inverse Simpson, and Shannon index values in patients with and without CDI converged.

We further assessed the α-diversity of fecal samples in patients with and without posttransplant CDI after stratifying by clinical variables, in particular those that were significantly associated with development of CDI (*P < *0.05) in univariable analyses. Among patients with Child-Pugh class C liver disease at the time of LT, α-diversity index values appeared to be similar in patients without CDI compared to those with CDI (Fig. S2). Conversely, in patients with Child-Pugh class A/B liver disease, those who developed posttransplant CDI compared to controls had lower Chao, Inverse Simpson, and Shannon index values pretransplant and at months 1, 3, 6, and 12 posttransplant. Reductions in the Inverse Simpson index were similarly more prominent at these time points in patients without a bile leak who developed CDI. In patients who developed a bile leak after LT, α-diversity index values were similar or slightly reduced in patients with and without CDI.

10.1128/msphere.00361-22.2 FIG S2 Differences in mean α-diversity (Chao, Inverse Simpson, Shannon) from pretransplant to 1 year posttransplant in fecal samples from LT patients with CDI, stratified by clinical variables. (A to F) Clinical variables that were found to be significantly associated with development of CDI after LT included Child-Pugh Class C liver disease (Chao [A], Inverse Simpson [C], and Shannon [E]) and biliary leak (Chao [B], Inverse Simpson [D], and Shannon [F]). Download FIG S2, TIF file, 2.8 MB. Copyright © 2022 Gomez-Simmonds et al. 2022 Gomez-Simmonds et al. https://creativecommons.org/licenses/by/4.0/ This content is distributed under the terms of the Creative Commons Attribution 4.0 International license.

**FIG 3:**
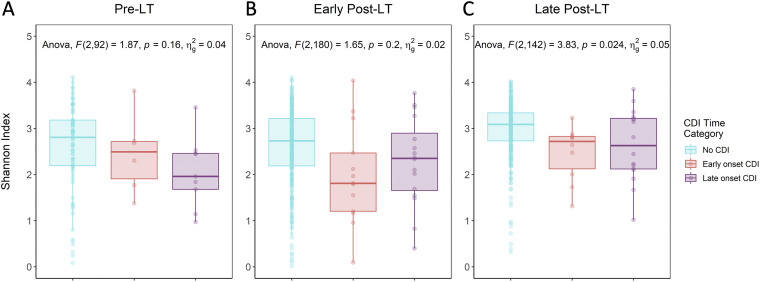
Mean Shannon α-diversity of fecal samples in patients with early versus late posttransplant C. difficile infection (CDI) and no CDI. (A, B) In the pretransplant (A) and early posttransplant (B) (≤30 days) sampling time points, no significant difference in Shannon α-diversity was observed between controls and patients with early- or late-onset CDI. (C) During the late posttransplant period (>30 days), Shannon α-diversity differed across the three groups (analysis of variance [Anova]; *P = *0.02), with the greatest difference observed between patients without CDI and those with late-onset CDI (Tukey *post hoc* test; *P = *0.05).

Last, we examined α-diversity of the intestinal microbiome in patients with early-onset (within 30 days) versus late-onset posttransplant CDI and further compared them to patients without CDI in order to determine whether microbial diversity may play a different role in the development of CDI at these time points (Fig. 3). On average, Shannon index values were lower in patients with both early and late posttransplant CDI compared to patients without CDI at all time points. Differences in Shannon index values significantly differed across categories in fecal samples collected during the late post-LT period only; adjusted pairwise analysis revealed a significant difference between patients with late-onset CDI and controls (Tukey *post hoc* test *P = *0.05).

### Differences in intestinal microbial composition in LT recipients with and without CDI.

**FIG 4:**
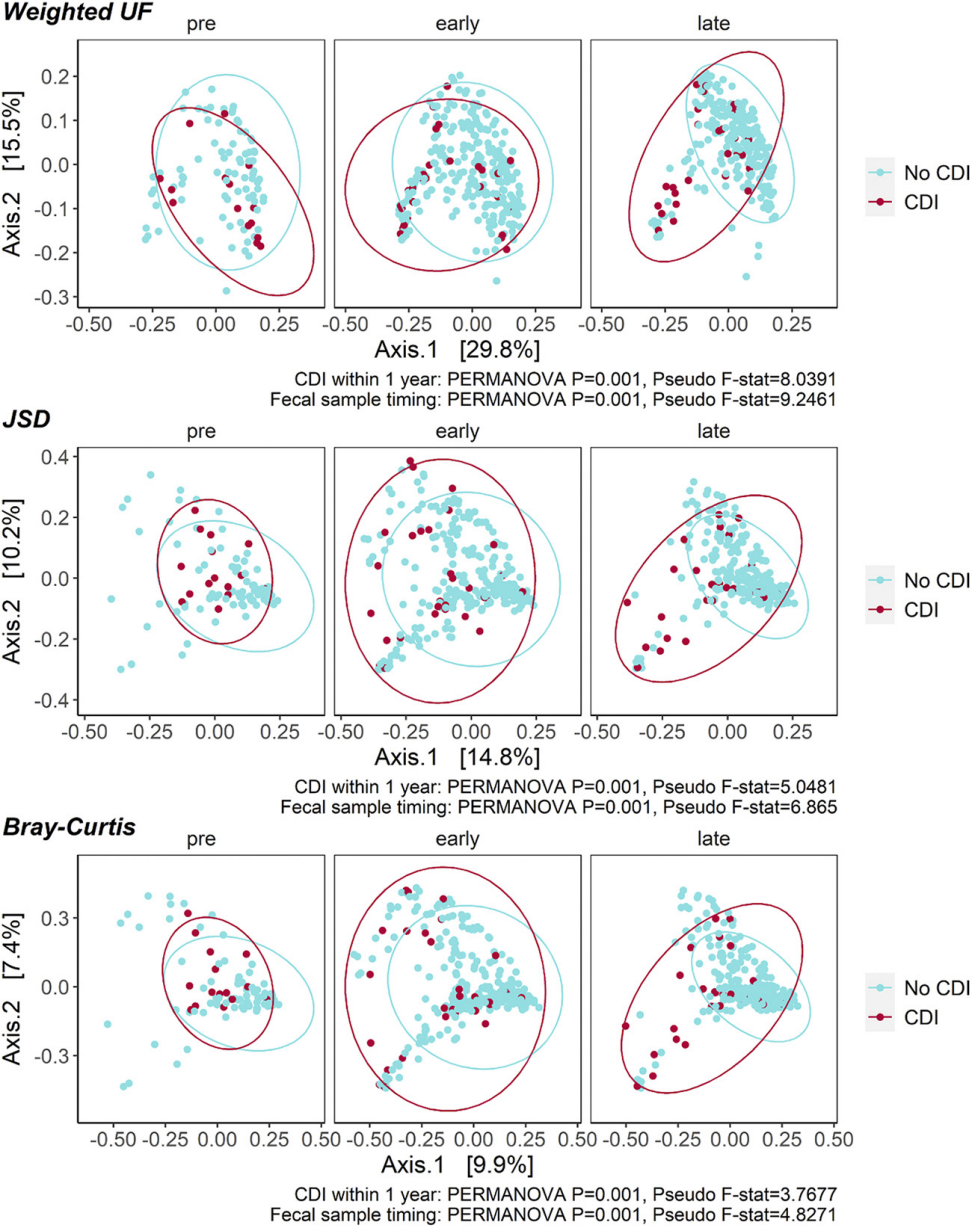
β-Diversity across sampling time points in patients with versus without *C. difficile* infection (CDI) and in patients with early versus late-onset posttransplant CDI. Principal-coordinate analysis (PCoA) based on weighted Unifrac (UF), Jensen-Shannon divergence (JSD), and Bray-Curtis β-diversity distances in fecal samples collected from patients with and without CDI revealed a shift in fecal microbiota at late-transplant sampling time points but not pretransplant or early posttransplant. PERMANOVA, permutational multivariate analysis of variance.

**FIG 5:**
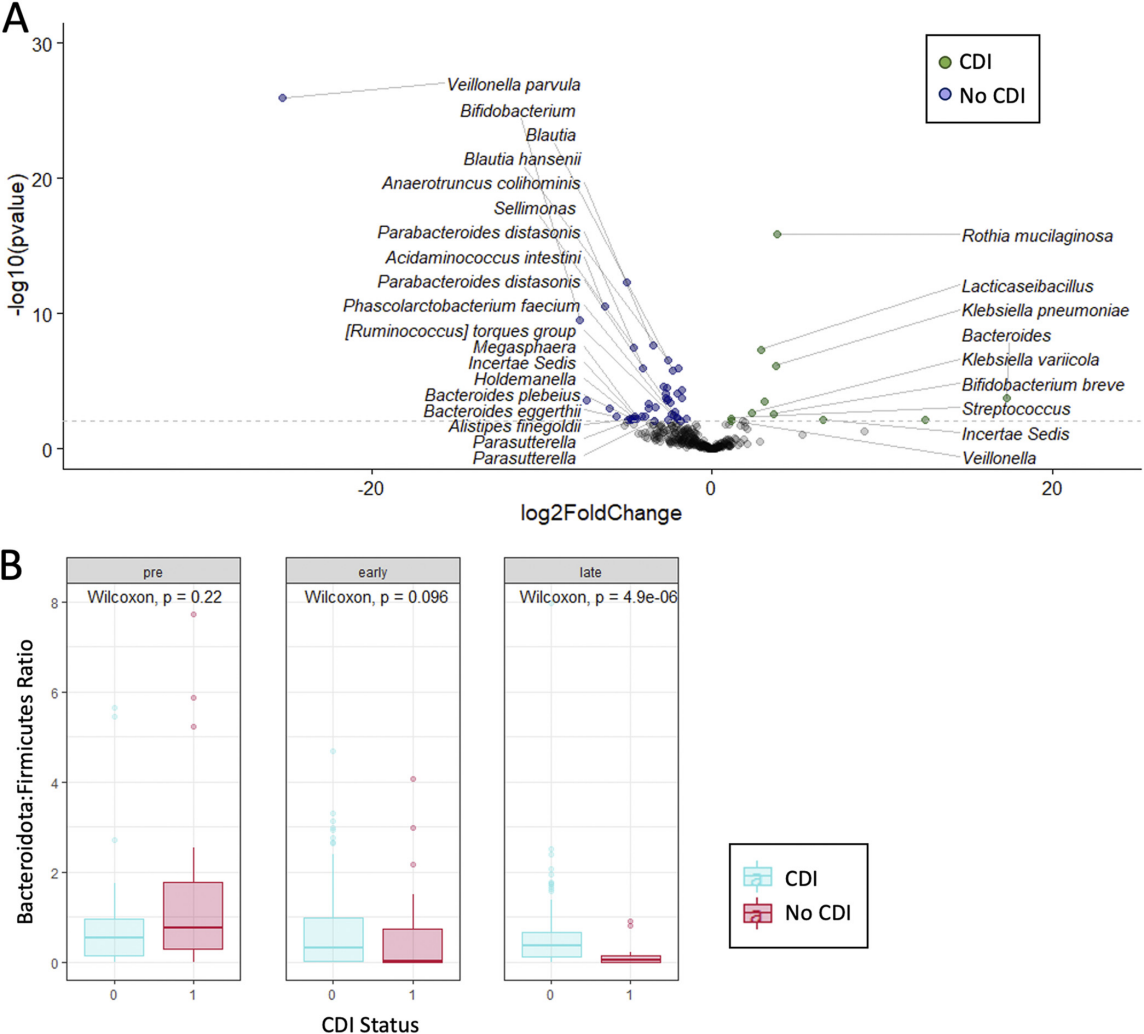
Differential abundances of taxa in fecal samples from patients with C. difficile infection (CDI) versus patients who did not develop CDI. (A) Differences in intestinal microbial composition in patients with CDI versus controls adjusted for sampling time point were assessed using a volcano plot to identify specific taxa that were differentially abundant in these two groups across all fecal samples. (B) Comparison of the *Bacteroidota*:*Firmicutes* ratio in patients with CDI versus controls in fecal samples collected during the pretransplant, early (≤30 days), and late (>30 days) posttransplant periods demonstrated a reduction in patients with CDI at all time points, although the difference was statistically significant in late posttransplant samples only.

We used principal-coordinate analysis (PCoA) based on weighted UniFrac, Jensen-Shannon Divergence (JSD), and Bray-Curtis β-diversity distances among fecal samples collected from patients with and without CDI to assess differences in microbial composition pretransplant and during the early (≤30 days) and late (>30 days) posttransplant periods (Fig. 4). All β-diversity metrics differed significantly based on both sampling time point and in CDI patients versus controls, most prominently later in the posttransplant course. To assess differences in intestinal microbial composition in patients with CDI versus controls, we used DADA2 to compare microbial community composition based on the relative abundance of amplicon sequence variants (ASVs) assigned to particular taxa across groups, adjusting for sampling time point (Fig. 5A). Samples from patients with CDI were enriched in Rothia mucilaginosa, Lacticaseibacillus spp., and *Proteobacteria* such as Klebsiella pneumoniae and Klebsiella variicola. Conversely, patients who did not develop CDI had increased abundance of Veillonella parvula, Bifidobacterium spp., members of the *Lachnospiraceae* family, and other taxa. In further exploring the relationship of taxonomic groups to each other with respect to CDI risk, we found the ratio of *Bacteroidota*:*Firmicutes* to be decreased in patients who developed CDI compared to controls, although this difference was significantly different in fecal samples collected during the late posttransplant period only (Fig. 5B).

### Relative abundance of C. difficile in fecal samples.

Based on 16S rRNA sequencing, we identified three C. difficile ASVs matching reference sequences in the Silva database; one of these ASVs was identified in all C. difficile-positive samples and was used in an analysis of C. difficile carriage. This C. difficile ASV was detected in any available fecal sample from 68 of 197 (35%) patients, including 10 of 18 (56%) of patients with CDI and 58 of 179 (32%) of patients without CDI (*P = *0.049). However, C. difficile was detected in ≥1 sample in only 11 of 166 patients with multiple samples, suggesting colonization was transient in most patients. Seven patients had evidence of persistent carriage for ≥3 months, none of whom developed CDI. Similarly, nine patients had high relative abundance of C. difficile (>1%) but did not develop clinical evidence of CDI. In patients who developed CDI after LT, C. difficile was detected in 7 of 9 (78%) fecal samples collected within 7 days before or after positive testing for CDI and 8 of 15 (53%) samples collected within 30 days. In 8 of 10 (80%) patients with available samples, C. difficile was not detectable in the first sample collected ≥7 days after positive testing, presumably due to effective antibiotic treatment.

We also compared detection of C. difficile based on 16S rRNA sequencing with PCR for toxigenic C. difficile for 150 of 576 stool samples collected from 87 patients without CDI. In 21 stool samples from 15 (8%) patients in which C. difficile was detected by 16S rRNA sequencing, PCR testing revealed the presence of the cytotoxin B gene *tcdB*, indicating that colonization with toxigenic C. difficile occurred in LT patients who did not develop clinical CDI. Two of these patients had high relative abundance of C. difficile; in patient 42, toxigenic C. difficile was detected in five separate stool samples collected pretransplant and at week 1 and months 3, 6, and 12 posttransplant. Additional review of clinical records does not indicate that this patient experienced any diarrheal illness nor need for antibiotic therapy at any point after LT. Toxigenic C. difficile was also detected by PCR in four stool samples without C. difficile identified by 16S rRNA sequencing. In 16 stool samples collected from 15 patients in which C. difficile was identified by 16S rRNA sequencing, *gluD* only was detected by PCR, suggestive of colonization with nontoxigenic C. difficile in these patients.

Finally, we compared the community composition of fecal samples from patients with CDI versus patients who had toxigenic C. difficile detected in by PCR but did not develop clinical infection in order to identify enrichment of potentially protective taxa in the latter group (Fig. S3). Interestingly, while Enterococcus faecium was enriched in patients with CDI, an ASV corresponding to a non-faecium Enterococcus had increased abundance in patients who did not develop infection. Control patients with PCR-detected toxigenic C. difficile were also noted to have increased abundance of Acidaminococcus intestini, Bacteroides spp., and members of the *Lachnospiraceae* and *Ruminococcus* families.

10.1128/msphere.00361-22.3 FIG S3 Volcano plot of differential abundances of taxa in fecal samples from patients with C. difficile infection (CDI) versus patients with toxigenic C. difficile detection who did not develop CDI. We compared the microbial community composition based on based on the relative abundance of amplicon sequence variants (ASVs) assigned to particular taxa in fecal samples from patients who developed posttransplant CDI versus controls who had C. difficile toxin detected in by PCR to identify potentially protective taxa. Download FIG S3, TIF file, 2.3 MB. Copyright © 2022 Gomez-Simmonds et al. 2022 Gomez-Simmonds et al. https://creativecommons.org/licenses/by/4.0/ This content is distributed under the terms of the Creative Commons Attribution 4.0 International license.

## DISCUSSION

In this study, we sought to identify clinical and microbiome factors associated with increased risk for CDI in an established, longitudinal cohort of LT recipients, 10% of whom developed CDI within 1 year of transplant. Patients with higher Child-Pugh scores at the time of LT and who developed postoperative biliary leak and/or were rehospitalized with need for antibiotics were significantly more likely to develop CDI within the first year posttransplant. Importantly, we found patients with posttransplant CDI to have significantly reduced intestinal α-diversity, most saliently at the 1 month posttransplant sampling period and in those who developed CDI during the late posttransplant period. We also noted significant differences in microbial composition (β-diversity) between patients with and without CDI and across sampling time points, particularly at later sampling time points. Taken together, these findings suggest that both clinical and intestinal microbiome factors play an important role in determining CDI risk in these patients and may vary over the posttransplant course.

Previous animal and human studies demonstrate that LT recipients experience marked perturbations in intestinal microbial communities, which may in turn affect posttransplant outcomes. In patients with end-stage liver disease, loss of microbial diversity as well as reductions in putatively beneficial bacteria and increases in pathogenic bacteria such as *Enterobacteriaceae* and *Enterococcus* have been shown to worsen at the time of transplant with partial recovery in subsequent months (22–24). Continued dysbiosis has been linked to posttransplant complications such as acute rejection, infection, and poor cognition (23, 24). In a previous study of this LT cohort, we found posttransplant MDRO colonization to be linked to changes in intestinal α-diversity, including during the pretransplant period (8), which was in turn an important predictor of subsequent infection (9). We also found differences in microbiome parameters across clinical subgroups of LT recipients. This study expands what is known about the relationship between LT and posttransplant infection risk by identifying specific microbiome factors implicated in posttransplant development of CDI in this high-risk patient population.

Decreases in α-diversity seen in LT patients may be further affected by antibiotic use, particularly in those with posttransplant complications such as biliary leak. Here, we found that α-diversity (by the Shannon, Chao, and Inverse Simpson indices) was significantly decreased in patients with posttransplant CDI at 1 month posttransplant, while Inverse Simpson and Shannon α-diversity remained significantly lower through month 6. This suggests that improvements in the number of taxa preceded sample evenness. Over time, increases in intestinal α-diversity seen in all groups may have served a protective role, reducing the risk of CDI in this population in later months. However, patients who developed late posttransplant CDI appeared to have persistent reductions in Shannon α-diversity in samples collected >30 days after transplantation, pointing to an ongoing role for microbial diversity in determining CDI risk later in the posttransplant course.

Although we had a limited ability to evaluate subgroup-level differences in intestinal α-diversity parameters due to our small sample size, we also identified notable trends in specific clinical subgroups. Interestingly, in patients with clinical markers for less severe pretransplant liver disease (Child-Pugh classes A and B) and no posttransplant development of biliary leak, α-diversity was decreased in those who developed posttransplant CDI compared to those who did not. Conversely, α-diversity parameters overlapped or were unexpectedly higher in patients with posttransplant CDI who had more severe liver disease (Child-Pugh class C liver disease and/or development of biliary leak). This suggests that in patients who are more clinically ill, changes in α-diversity may play an overall less important role in determining susceptibility to CDI than other risk factors. Indeed, in a subset of LT patients in our cohort, we were also not able to identify clinical or microbiome factors that contributed to CDI risk, for example those who developed CDI despite high or increasing intestinal α-diversity. In addition to microbial factors, the degree of posttransplant immune dysregulation may also have played a role in determining posttransplant CDI risk. Here, a “multihit” hypothesis may be at play, in which risk of exposure, changes in intestinal microbiota, and immunologic status collectively contribute to CDI susceptibility and resistance posttransplant. Further studies are needed to explore the effect of posttransplant immune dysregulation on CDI risk and investigate the relationship between clinical risk factors, immune system function, and the intestinal microbiome.

Previous studies have found specific bacterial taxa to be enriched in patients with CDI, such as *Peptostreptoccocaceae*, *Proteobacteria*, and *Enterococci*, while taxa such as *Bacteroides* and *Firmicutes* appear to be diminished in rodent and human studies (10, 12, 15, 16, 25–27). Notably, similar taxa may be seen in C. difficile-negative nosocomial diarrhea. These microbes have been postulated to enhance biological niches conducive to C. difficile germination and invasion, stimulate and dysregulate host immune functions, and interfere with recovery of normal flora to enable CDI and persistence. In this study, we found patients with CDI to have increased abundance of *Proteobacteria*, as well as R. mucilaginosa and Lacticaseibacillus spp., and to lack putatively protective taxa such as *Bifidobacterium* spp. and *Lachnospiraceae*. Consistent with prior studies (16, 27), the *Bacteroides*:*Firmicutes* ratio was significantly reduced in patients with CDI in samples collected during the late posttransplant period. We also identified a subset of patients (8%) without clinical evidence for CDI who were colonized with toxigenic C. difficile based on identification of the *tcdB* gene in fecal samples. These patients may have had additional protective factors that prevented development of symptomatic CDI, including increased abundance of A. intestini and members of the *Lachnospiraceae* and *Ruminococcus* families. Further studies investigating microbial factors that could reduce colonization or prevent development of symptomatic CDI in this patient population are warranted.

Our study had several limitations. While a relatively large proportion of LT patients in our cohort developed CDI, the number of cases was small, limiting our ability to detect subtle differences between patient groups and conduct subgroup analyses. This is particularly relevant in this patient population, in which variability in underlying liver disease severity and the posttransplant clinical course can likely substantially alter microbiome parameters and CDI risk. Moreover, fecal sample collection did not occur within prespecified time intervals of positive testing for C. difficile, and thus we were able to describe CDI onset only within the broader context of pre- and posttransplant microbiome changes. This may have particularly limited assessment of CDI-associated taxa. Although for most cases, stool samples were available within 30 days of positive clinical testing, in some cases patients may have commenced treatment for C. difficile prior to sample collection. Diagnosis of CDI was based on clinical PCR-based testing, which may have a limited ability to distinguish between patients with C. difficile colonization and infection, particularly in a patient population in which diarrhea is common. Additional testing for high-risk strains, such as the NAP1/B1/027 strain, was also not available. Because we only collected fecal samples for the purposes of this study, we were not able to test immune and inflammatory factors in our cohort, which likely play an important role in CDI risk in SOT recipients and may contribute to shaping microbial communities and sustaining dysbiosis. Finally, we had incomplete records of the antibiotics used in the outpatient setting and during hospitalization at other institutions, limiting our analysis to inpatient antibiotic exposure during the initial transplant and any subsequent posttransplant hospitalizations.

In summary, we found both clinical and microbiome factors to contribute to the development of CDI in a cohort of patients undergoing LT. While CDI frequently developed during the early posttransplant period, patients with reductions in α-diversity later in the posttransplant course may have ongoing susceptibility to development of CDI. Although recovery of intestinal microbial diversity over the first year posttransplant is likely to be protective against CDI during later posttransplant periods, robust antibiotic stewardship practices, particularly during the late posttransplant period and posttransplant rehospitalizations, may reduce CDI risk. Patients with high-risk features such as more severe liver disease, development of biliary leak, and extensive antibiotic use may also benefit from preventive measures to preserve intestinal diversity. While the role of FMT in CDI prevention is unclear, these patients may benefit from future microbiome modulatory therapies. Finally, this study suggests a need to further examine clinical, immunologic, and microbiome factors associated with the development of CDI in specific clinical contexts following LT to identify additional modifiable risk factors to prevent CDI in this high-risk patient population.

## MATERIALS AND METHODS

### Patient population.

Our approach to patient recruitment and sample collection has been described previously (9). Briefly, adult patients (>18 years old) listed for LT at a tertiary care hospital between March 2014 and January 2017 were approached for informed consent. Enrollees were asked to provide fecal samples every 6 months prior to transplantation and at week 1 and months 3, 6, and 12 posttransplant. Only subjects who provided at least one fecal sample were included in the analysis. Approval for the study protocol was obtained from the Columbia University Irving Medical Center (CUIMC) Institutional Review Board (approval AAAM7704).

### Clinical data extraction and definitions.

We extracted data on patient demographics, comorbidities, LT-related clinical parameters such as liver disease etiology and severity at time of transplant, peri- and postoperative complications, and inpatient antibiotic use from electronic medical records as described previously (8, 9). Comorbidities were summarized using the Charlson Comorbidity Index (CCI) score based on admission documentation from the transplant hospitalization (28). Severity of liver disease at the time of LT was reported using the model for end-stage liver disease-Na (MELD-Na) and Child-Pugh score and class (A, B, and C). Complications included prolonged ischemic time, bleeding, biliary complications such as development of leak or stricture, and need for intensive care unit (ICU) readmission during the initial LT hospitalization, as well as hospital readmission, rejection episode as confirmed by liver biopsy, and death within 1 year of LT. All inpatient antibacterial use was collected and classified by drug class; however, oral vancomycin and oral or intravenous (IV) metronidazole, which were recommended for treatment of CDI by our institutional guidelines during the study period, were excluded from the analysis. Antibiotic exposure was calculated based on aggregate number of days exposed for each individual antibiotic during the first year after LT.

For the purposes of this study, we also collected data on all available pre- and posttransplant clinical testing results for C. difficile. CDI was defined by positive stool PCR testing using the Xpert C. difficile assay (Cepheid). Notably, at our institution, CDI testing is performed only on unformed stool, consistent with established guidelines (21). The incident episode of CDI for each patient was defined as occurring pretransplant or during the early (within 30 days) or late (>30 days) posttransplant periods. Fecal samples were coded based on collection date as pretransplant, week 1 (days 0 to 14), month 1 (days 15 to 60), month 3 (days 61 to 135), month 6 (days 136 to 270), and month 12 (days 271 to 395) posttransplant. Additional clinical data collected that was relevant to the CDI diagnosis included vital signs, peak and nadir WBC count, serum creatinine and albumin levels, and ICU admission within 48 h of positive testing. CDI episodes were categorized as nonsevere, severe, and fulminant based on 2017 treatment guidelines (21), in which clinical markers for severe infection include WBC count ≥15,000 cells/mL and/or a serum creatinine level ≥1.5 mg/dL, and fulminant infection is defined by development of hypotension, shock, ileus, and/or megacolon. CDI recurrence was defined as positive testing at least 2 weeks after completion of previous treatment course with documented resolution of prior symptoms.

### Microbiological procedures, sequencing, and microbiome analysis.

Rectal swab and fecal samples underwent DNA extraction followed by 16S rRNA V3/V4 amplification and sequencing on Illumina MiSeq or HiSeq platforms as described previously (8). Negative controls (nuclease-free water) were included for each 16S rRNA amplification and library prep batch. 16S rRNA sequences were processed using DADA2 version 1.10.1 in R statistical software version 3.6.1 (29). Sequences were quality filtered and trimmed, after which chimeric reads were removed, and the resulting reads were error corrected. Quality-controlled reads were used to define unique ASVs, which were then clustered at 97% sequence identity. Taxonomic classification was performed using the Silva database (30), and mitochondrial reads and those unassigned at the phylum level were removed. ASVs with an average relative abundance of <0.005% across all samples were filtered using the *phyloseq* version 1.19.1 package in R (31). Within-sample α-diversity measures for species abundance and richness (Chao, Shannon, Inverse Simpson) were then calculated using *phyloseq.* Based on α-diversity rarefaction, we applied a minimum cutoff of 7,500 counts for inclusion in the analysis. For samples in which C. difficile was detected using 16S rRNA sequencing, we also performed PCR targeting the *tcdB* gene, which encodes cytotoxin B produced by all toxigenic strains, to differentiate between toxigenic (*tcdB-*positive) and nontoxigenic (*tcdB-*negative) C. difficile. The gene encoding glutamate dehydrogenase (*gluD*) was also targeted to confirm detection of C. difficile. Established PCR primers for *tcdB* (32) and *gluD* (33) were used.

### Statistical analysis.

Clinical characteristics of patients with and without episodes of CDI were assessed using univariable models. Categorical variables were compared using χ^2^ or Fisher’s exact tests for expected frequencies below 5; continuous variables were compared using *t* tests or Wilcoxon rank sum tests as appropriate after testing for normality using the Shapiro-Wilk test in conjunction with graphical data representations (histograms, qq-plots) in RStudio version 1.4.1106. For all comparisons, statistical significance was defined by *P* < 0.05. For variables found to be statistically significantly associated with development of posttransplant CDI, we constructed a directed acyclic graph to model the relationship between variables (Fig. S1) (34).

We also assessed univariable associations between CDI status and intestinal microbiome α-diversity (Shannon, Chao, Inverse Simpson) for fecal samples collected pretransplant and at week 1, month 1, month 3, month 6, and month 12 posttransplant using *t* tests after confirming normality. For some analyses, the sampling time points were further grouped as follows: pretransplant, early posttransplant (≤30 days), and late posttransplant (>30 days). Mean Shannon index values were compared among patients with early and late posttransplant CDI and controls using analysis of variance (ANOVA). *Post hoc*, between-group comparisons were performed using the Tukey test. Last, stratified analyses were performed for clinical variables with univariable *P* < 0.05 including Child-Pugh class A/B versus C and posttransplant development of biliary leak.

To test differences in β-diversity across groups, PCoA in *phyloseq* and permutational multivariate analysis of variance (PERMANOVA) tests in *vegan* version 2.4-4 (35) were used based on weighted UniFrac, JSD, and Bray-Curtis distance matrices. *DESeq2* version 1.14.1 (36) was used to identify differentially abundant bacterial taxa.

### Data availability.

Sequencing data are publicly available through the NCBI Sequencing Read Archive (SRA) (BioProject accession number PRJNA522306).
