# Supplementary figures and images for: Intestinal Dysbiosis and Risk of Posttransplant Clostridioides difficile Infection in a Longitudinal Cohort of Liver Transplant Recipients
Source: mSphere. 2022 Sep 22;7(5):e00361-22. doi: 10.1128/msphere.00361-22 (PMC9599498; doi:10.1128/msphere.00361-22)

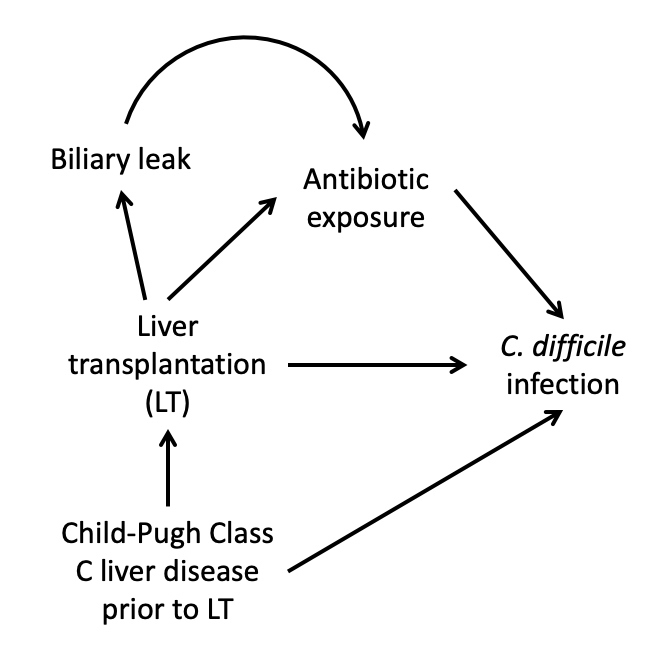

Supplement: FIG S1 [file msphere.00361-22-s0001.tif]

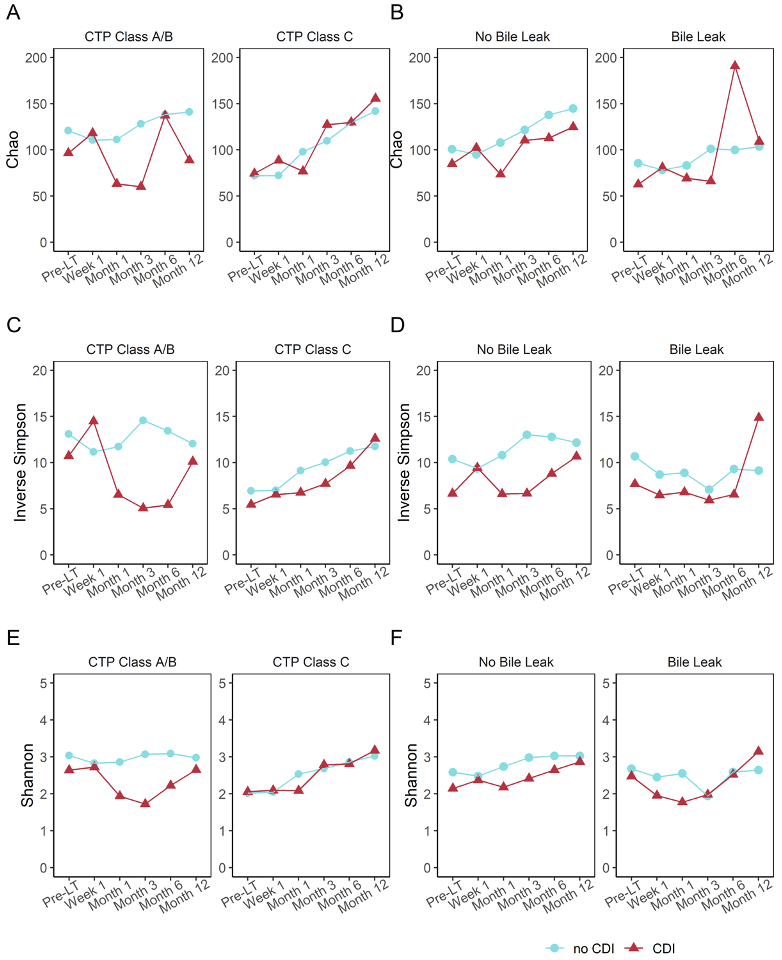

Supplement: FIG S2 [file msphere.00361-22-s0002.tif]

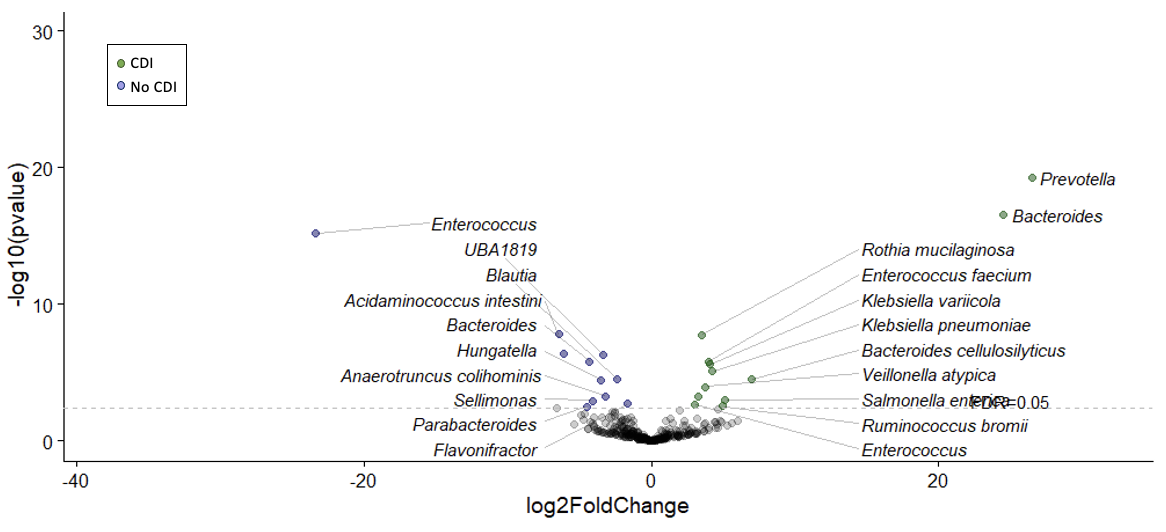

Supplement: FIG S3 [file msphere.00361-22-s0003.tif]
